# Benign esophageal schwannoma: a brief overview and our experience with this rare tumor

**DOI:** 10.1186/s40792-017-0369-0

**Published:** 2017-08-31

**Authors:** Kazuki Moro, Masayuki Nagahashi, Kotaro Hirashima, Shin-ichi Kosugi, Takaaki Hanyu, Hiroshi Ichikawa, Takashi Ishikawa, Gen Watanabe, Emmanuel Gabriel, Tsutomu Kawaguchi, Kazuaki Takabe, Toshifumi Wakai

**Affiliations:** 1 Division of Digestive and General Surgery, Niigata University Graduate School of Medical and Dental Sciences, 1-757 Asahimachi-dori, Chuo-ku, Niigata City, Niigata 951-8510 Japan; 2 Division of Molecular and Diagnostic Pathology, Niigata University Graduate School of Medical and Dental Sciences, 1-757 Asahimachi-dori, Chuo-ku, Niigata City, 951-8510 Japan; 3 Department of Surgical Oncology, Roswell Park Cancer institute, Buffalo, NY 14263 USA; 4 Department of Surgery, University at Buffalo Jacobs School of Medicine and Biomedical Sciences, the State University of New York, Buffalo, NY 14203 USA

**Keywords:** Esophagus, Schwannoma, Enucleation, Cervical approach

## Abstract

**Background:**

Benign esophageal tumors are uncommon, comprising approximately 2% of esophageal tumors. Esophageal schwannomas constitute an even rarer entity, with few cases reported in the literature.

**Case presentation:**

We present a 66-year-old male who was referred for dysphagia. A computed tomography scan showed a well-demarcated, enhancing, and homogenous esophageal tumor measuring 50 mm. The tumor was hypermetabolic on positron emission tomography, and an endoscopic ultrasound-guided fine needle aspiration demonstrated the presence of benign spindle cells. We performed an uncomplicated, simple, tumor enucleation through a cervical approach. Histology revealed spindle-shaped cells in a fasciculated, disarrayed pattern. Immunohistochemistry demonstrated positive staining for S-100 protein and negative staining for KIT, CD34, desmin, and α-smooth muscle actin. These findings were consistent with a benign esophageal schwannoma.

**Conclusions:**

We report our experience with esophageal schwannoma, a rare but benign diagnosis of the esophagus.

## Background

Approximately 2% of all esophageal tumors are benign primary tumors of the esophagus, and these are most commonly located in the upper thoracic esophagus [1, 2]. Many patients are asymptomatic and are incidentally found to have benign esophageal tumors. The most common symptom is mild to moderate dysphagia; however, dyspnea frequently occurs and has been increasingly reported as a presenting symptom [2–5]. In contrast, severe or complete esophageal obstruction from benign tumors has not been previously reported, and acute surgical treatment is typically unnecessary [6]. Over 80% of benign esophageal tumors are leiomyomas [2, 7].

Esophageal schwannomas, in contrast, are rare benign neurogenic tumors reported to have no favorite site [8]. Typically originating in the mediastinum, due to their featureless and nonspecific characteristics, esophageal schwannoma is difficult to diagnose by standard imaging techniques, including computed tomography (CT) and magnetic resonance imaging (MRI) scans. Recently, 18-Fluorodeoxyglucose positron emission tomography (FDG-PET) used in conjunction with conventional CT and MRI was reported to aid in the diagnosis of esophageal tumors [9]. High FDG uptake is generally found in malignant tumors. While esophageal schwannomas are benign tumors, it shows a hypermetabolic appearance on FDG-PET [10, 11]. Furthermore, endoscopic ultrasound-guided fine needle aspiration (EUS-FNA) can be used to help establish the pathologic diagnosis, but this technique has limited accuracy [12].

Surgical treatment for benign esophageal tumors arising from the submucosal layer is controversial. In general, surgical resection should be considered for patients with benign esophageal tumors which are large, symptomatic, or increasing in size [11]. Additionally, suspected malignant potential is usually considered to be an indication of an extended operation such as total esophagectomy. In this case report, we describe our surgical experience with benign esophageal schwannomas, with a brief review of other reported cases in the literature.

## Case presentation

**Fig. 1:**
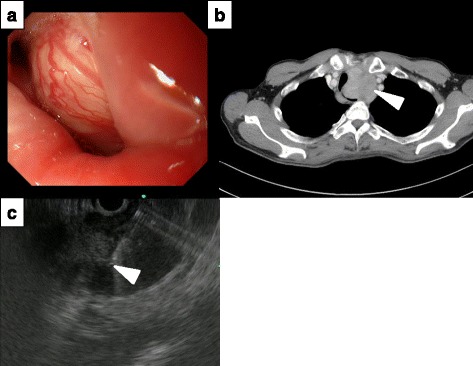
The examination image before the operation. **a** Endoscopy revealed an approximately 5.0 cm esophageal submucosal tumor, which was located 25 cm from the incisors. It occupied the majority of the esophageal lumen. **b** Computed tomography revealed a large isodense tumor of the esophageal wall in the upper mediastinal space (*white arrow*). **c** Endoscopic ultrasonography showed a large tumor (*white arrow*) of low echogenicity in the submucosa layer

A 66-year-old male was referred to Niigata University Hospital after being found to have tracheal deviation on an X-ray obtained to diagnose the cause of dysphagia. He had a history of smoking and drinking for 46 years. A physical examination showed no significant findings, and laboratory tests, including serum tumor markers such as CEA and CA19-9, were normal. Endoscopy revealed an approximately 50 mm esophageal submucosal tumor, which was located 25 cm from the incisors (Fig. 1a). A chest CT scan showed a well-demarcated, enhancing homogeneous tumor measuring 51 × 41 mm in the upper third of the esophagus (Fig. 1b). No regional lymph node enlargement was observed. Endoscopic ultrasonography (EUS) showed a large tumor of low echogenicity in the esophageal wall, originating in the submucosa layer (Fig. 1c). EUS-FNA-mediated histopathological examination revealed a proliferation of spindle-shaped cells in a fasciculated, disarrayed architecture, with nuclei in a palisading pattern, followed by a preoperative diagnosis of esophageal schwannoma was made in this current case.

**Fig. 2:**
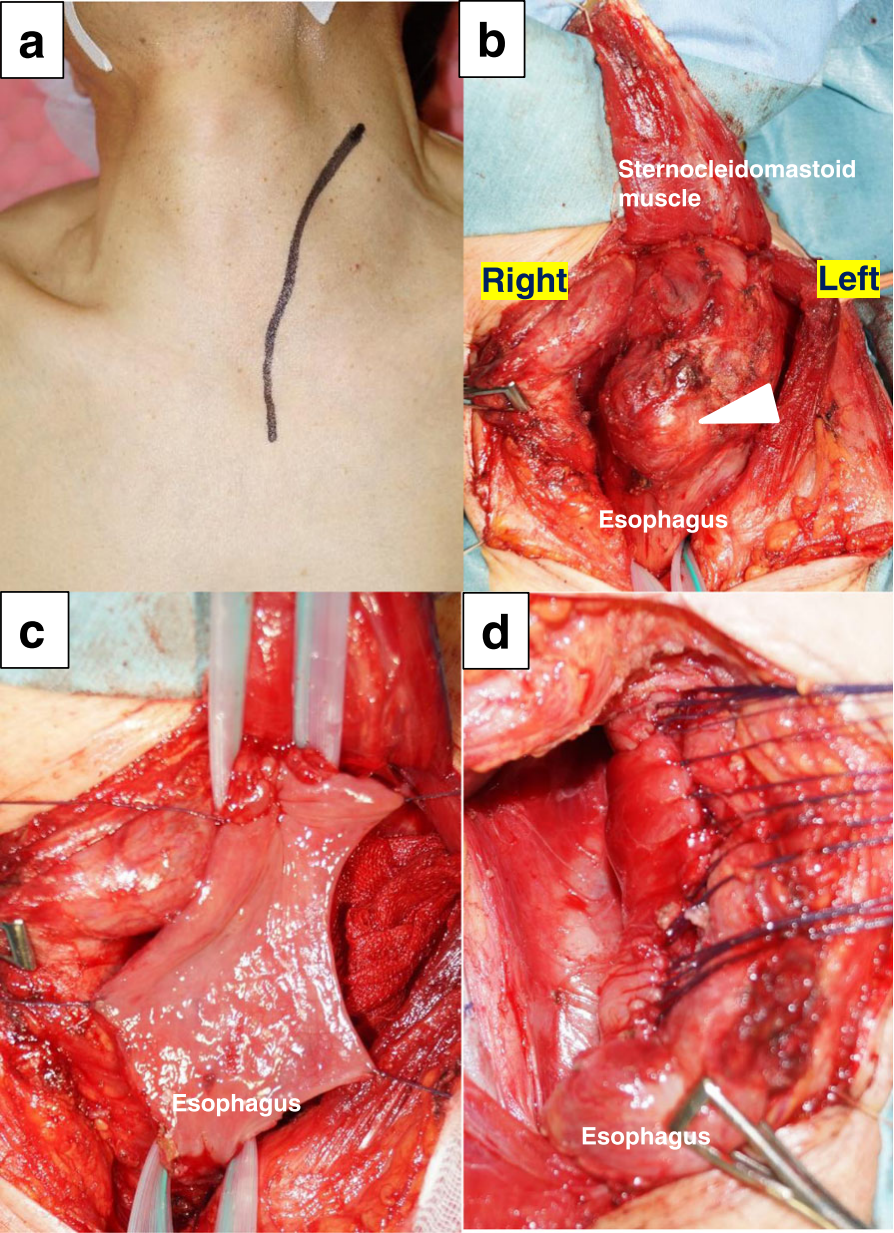
Surgical approach. **a** The skin incision used for the cervical approach. **b** The tumor was found close to the upper thoracic esophagus (*white arrow*). After dividing the left sternocleidomastoid muscle, the tumor was identified easily. **c** The esophagus was retracted at the proximal and distal side of the tumor. The tumor was then excised, including all layers of the esophageal wall. **d** The esophagus was closed transversely to include each layer

Given that the 50-mm tumor was located at the anterior wall in the upper third of the esophagus, we performed tumor enucleation using a cervical approach. We placed the skin incision in a longitudinal fashion along the anterior border of the sternocleidomastoid muscle extending to the sternal notch, which facilitates use of a combined transthoracic approach if needed (Fig. 2a). After transection of the left sternocleidomastoid muscle, the tumor was easily identified (Fig. 2b). After mobilization of the esophagus, the tumor was excised to include all esophageal layers (Fig. 2c). Although preservation of the mucosal layer with tumor enucleation is a less invasive technique, in our case, full-thickness excision was indicated because of dense adhesions between the tumor and the surrounding esophagus as well as thinning of the mucosal and muscular layers. We inserted an endoscope to act as a stent during transverse closure of the esophagus and performed a leak test that was negative (Fig. 2d). The anastomosis appeared healthy and well perfused, and we were careful not to injure the blood supply to the surrounding esophagus. Post-procedure, neither dehiscence nor stricture was found by fluoroscopy. Following this normal post-operative test result, we removed the nasogastric tube and permitted the patient to start eating.

**Fig. 3:**
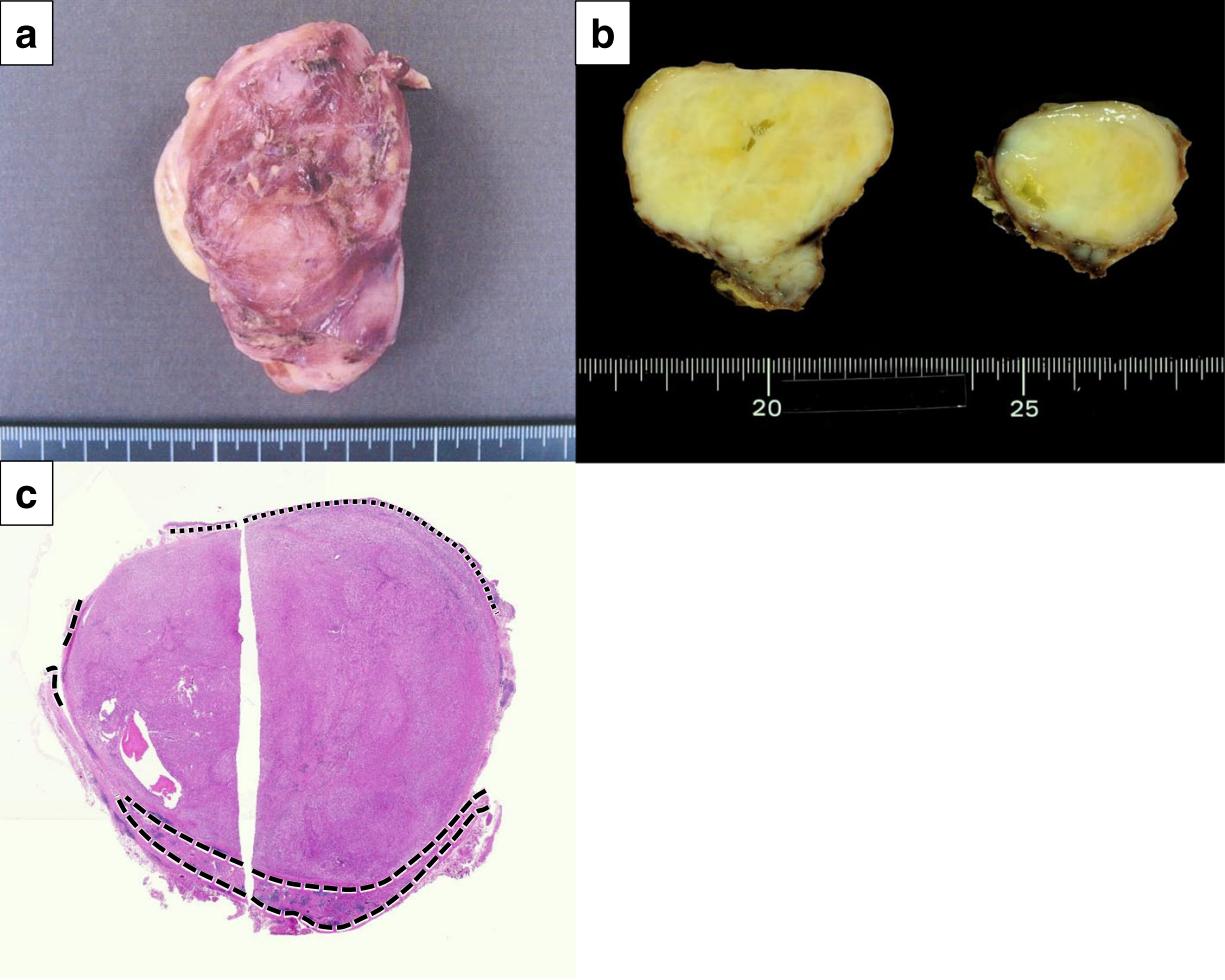
Macroscopic findings. **a** The resected tumor was 5.2 × 4.0 × 3.1 cm in size. **b** The cut surface on gross examination was yellowish and elastic. **c** The tumor originated in submucosa. The *narrow-dotted line* showed smooth muscle (muscularis mucosae). The *heavy-dotted line* showed cross-striated muscle (tunica muscularis). The mucosal and muscular layers were thinned due to the local compressive effects of the tumor

**Fig. 4:**
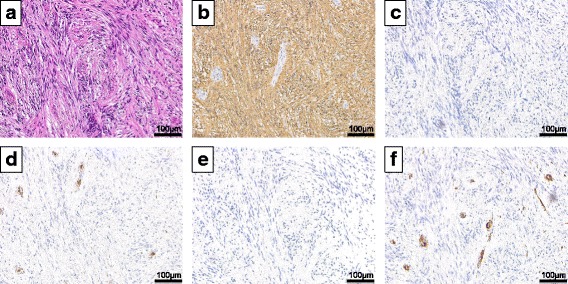
Histopathological findings. **a** Histopathological findings revealed spindle-shaped cells (hematoxylin and eosin stain, ×200). **b–f** Immunohistochemical staining of tissue sections with nuclei counterstained with hematoxylin. **b** The tumor showed cytoplasmic expression of S-100 (×200). **c** The tumor was negative for KIT (×200). **d** The tumor was negative for CD34 (×200). Vascular endothelial cell of the tumor was immunostained. **e** The tumor was negative for Desmin (×200). **f** The tumor was negative for α-SMA (×200). Vascular smooth muscle cells of the tumor were immunostained

The resected tumor measured 52 × 40 × 31 mm in size (Fig. 3a), and the cut surface on gross examination was yellowish and elastic (Fig. 3b). Consistent with the EUS which showed that the tumor was originating in the submucosa layer, pathological analysis confirmed that the submucosa was the originating layer (Fig. 3c). High-power photomicrographs of the tumor showed spindle-shaped cells in a fasciculated and disarrayed architecture (Fig. 4a). Immunohistochemically, the cells stained positive for S-100 protein (Fig. 4b), but were negative for KIT (Fig. 4c), CD34 (Fig. 4d), desmin (Fig. 4e), and α-smooth muscle actin (SMA) (Fig. 4f). This was consistent with the tumor being a benign esophageal schwannoma. The patient recovered without any post-operative complications and was discharged on the 12th post-operative day, which is standard for an open esophageal enucleation. There has been no evidence of recurrence over a current follow-up period of 3 years.

### Discussion

Esophageal schwannoma is one of the most common types of neurogenic tumor. Benign disease is uncommon, but malignant schwannoma is even more rare [13]. Although esophageal schwannoma is often difficult to diagnose preoperatively [14], an accurate preoperative diagnosis could lead to less invasive surgical treatment. Therefore, although this is a rare entity, it is important to suspect esophageal schwannoma by clinical examination and subsequent pathologic biopsy to establish an accurate preoperative diagnosis.

Regarding the presentation of esophageal schwannoma, while some patients are asymptomatic, symptoms generally correlate with tumor size due to the mass impinging upon surrounding structures, which can result in dysphagia, dyspnea, chest pain, pneumonia, or hemoptysis [5]. On review of our own retrospective series of four patients with esophageal schwannoma, including this current case, three patients presented with progressive dysphagia. Table 1 summarizes the characteristics of our patient series. Obtaining an accurate preoperative diagnosis of esophageal schwannoma is very challenging. EUS-FNA may be useful for both the diagnosis and management of this disease [15]. Although EUS-FNA may have several procedural risks, such as bleeding and infection, these risks are minimal [16]. In general, it is considered to be a safe, reliable, and accurate method for obtaining a tissue diagnosis in the evaluation of submucosal lesions of the gastrointestinal tract. In this case, we could make a preoperative diagnosis by EUS-FNA. Even if preoperative diagnosis was difficult to make, using operative rapid pathologic diagnosis method may also be useful.

**Table 1:** Characteristics of the patient series with esophageal schwannoma

	Case 1	Case 2	Case 3	Case 4
Age (years)	73	36	41	66
Gender	Female	Female	Male	Male
Chief complaint	Dysphagia	Dysphagia	Dysphagia	Asymptomatic
Past medical history	Hypertension	Appendicitis	Hemorrhoids	None
Tumor location	Upper third of the esophagus	Upper third of the esophagus	Middle third of the esophagus	Upper third of the esophagus
Tumor size (cm)	4.0 × 3.0 × 3.5	9.0 × 6.7 × 3.9	4.7 × 3.7 × 3.1	7.5 × 3.1 × 3.5
Lymph node involvement	Negative	Negative	Negative	Negative
PDG-PET	Accumulated	Accumulated	Accumulated	Not performed
EUS-FNA	Not performed	Not performed	Performed (did not establish diagnosis)	Performed (established diagnosis)
Surgical treatment	Enucleation	Esophagectomy	Enucleation	Enucleation

*EUS-FNA* endoscopic ultrasound-guided fine needle aspiration, *PDG-PET* 18-fluorodeoxyglucose positron emission tomography

While surgical resection offers radical treatment for esophageal schwannoma, the approach should be determined based on tumor size, location, and patient condition. Esophagectomy or local resection consisting of full-thickness excision and tumor enucleation are mainly performed. As a more radical approach, esophagectomy may lead to a high incidence of post-operative complications, such as recurrent laryngeal nerve paralysis, pulmonary compromise, or chylothorax [17, 18]. In contrast, local resection is a sufficient approach for the curative treatment of benign schwannomas and is less likely to result in serious morbidity [19]. Furthermore, tumor enucleation is quite technically feasible because the esophageal schwannoma does not usually involve all layers of the esophageal wall and is typically limited to the submucosa [20]. However, enucleation may not be a preferred approach for very large tumors because this has been associated with higher rates of esophageal stenosis [21].

When the tumor is located in the upper third of the esophagus as in this case, a cervical approach for enucleation has been reported [22, 23]. Conversely, a transthoracic approach presents more difficultly for resection of a tumor located in the cervical esophagus because of its deeper operative field and narrower working space. In this case, the tumor was located at the anterior wall in the upper third of the esophagus that we could address using the cervical approach. In our series of four patients with esophageal schwannoma, three patients underwent tumor enucleation and one patient underwent esophagectomy due to the excessive size of the tumor (90 × 67 mm).

Our case represents the first case report of tumor enucleation through a cervical approach. Recently, however, video-assisted thoracic surgery (VATS) has been used more, as this minimally invasive approach has been shown to result in less post-operative pain and shorter hospital length of stay than the open thoracotomy approach. However, in certain cases, such as in one of our patients, VATS may not provide exposure that is adequate for tumor access. In very large submucosal tumors, VATS may lead to an increased risk of mucosal injury during extensive submucosal dissection [24].

There are a few reported cases of malignant esophageal schwannoma [25, 26]. In these cases, regional lymph node dissection was performed, and the patients did not experience any recurrences. In contrast with radical lymph node dissection in esophageal cancer (adenocarcinoma or squamous cell carcinoma), lymph node dissection for esophageal submucosal tumors is controversial. When an esophageal submucosal tumor is suspected to be malignant based on concerning preclinical or radiographic findings (such as local invasion or enlarged suspicious lymph nodes), lymph node dissection should be considered. Radical esophagectomy with regional lymph node dissection may also be needed in certain cases to minimize potential recurrence. In this case, we achieved successful tumor enucleation for a large benign submucosal tumor; however, we emphasize that surgery to treat benign esophageal tumors, including schwannomas, should be performed on a case-by-case basis.

## Conclusions

Herein, we presented our experience with esophageal schwannoma and a current review of the literature. It is important to establish a diagnosis prior to resection as this may favor less invasive treatment. FNA via EUS can assist in the diagnosis when used in conjunction with cross-sectional imaging. When accurate diagnosis cannot be obtained preoperatively, intraoperative rapid pathologic diagnosis should be considered. Local resection including tumor enucleation is a therapeutic option with limited surgical stress and satisfactory clinical outcomes in patients with esophageal schwannoma, as was the case for each of the patients in our series.

## Abbreviations

CT: Computed tomography
EUS-FNA: Endoscopic ultrasound-guided fine needle aspiration
FDG-PET: 18-Fluorodeoxyglucose positron emission tomography
MRI: Magnetic resonance imaging
SMA: α-Smooth muscle actin
VATS: Video-assisted thoracic surgery
